# Crystal structure of 2,3,5,6-tetra­kis[(methyl­sulfan­yl)meth­yl]pyrazine^1^

**DOI:** 10.1107/S1600536814011246

**Published:** 2014-08-01

**Authors:** Tokouré Assoumatine, Helen Stoeckli-Evans

**Affiliations:** a CanAm Bioresearch Inc., 9-1250 Waverley Street, Winnipeg, Manitoba, R3T 6C6, Canada; b Institute of Physics, University of Neuchâtel, rue Emile-Argand 11, CH-2000 Neuchâtel, Switzerland

**Keywords:** crystal structure, tetrakis-substituted, pyrazine, sulfanyl-methyl derivative, inversion symmetry

## Abstract

The title compound, C_12_H_20_N_2_S_4_, synthesized by the reaction of 2,3,5,6-tetra­kis­(bromo­meth­yl)pyrazine with sodium methane­thiol­ate, crystallizes with a half -mol­ecule in the asymmetric unit. The whole mol­ecule is generated by inversion symmetry; the inversion centre being located in the centre of the pyrazine ring. The mol­ecule has an S-shaped conformation with two (methyl­sulfan­yl)methyl substituent arms directed above the plane of the pyrazine ring and two below. The C(H_3_)—S—C(H_2_)—C(aromatic) torsion angles are 70.47 (18) and −67.65 (17)°, respectively. In the crystal, mol­ecules are linked *via* weak C—H⋯S hydrogen bonds, forming chains along [001] and enclosing *R*_2_^2^(12) ring motifs. The chains are linked by further weak C—H⋯S hydrogen bonds, forming sheets lying parallel to (101).

## Related literature   

For syntheses of the starting reagent, 2,3,5,6-tetra­kis­(bromo­meth­yl)pyrazine, see: Ferigo *et al.* (1994 ▶); Assoumatine (1999 ▶); Assoumatine & Stoeckli-Evans (2014 ▶). For the crystal structures of similar sulfanylmethyl derivatives of pyrazine, such as two triclinic polymorphs of 2,3,5,6 tetra­kis­(naphthalen-2-ylsulfanylmeth­yl)pyrazine both possessing inversion symmetry, see: Pacifico & Stoeckli-Evans (2004 ▶), and for 2,3,5,6-tetra­kis­(phenyl­sulfanylmeth­yl)pyrazine, which also crystallizes in space group *P* 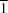 and possesses inversion symmetry, see: Assoumatine *et al.* (2007 ▶).
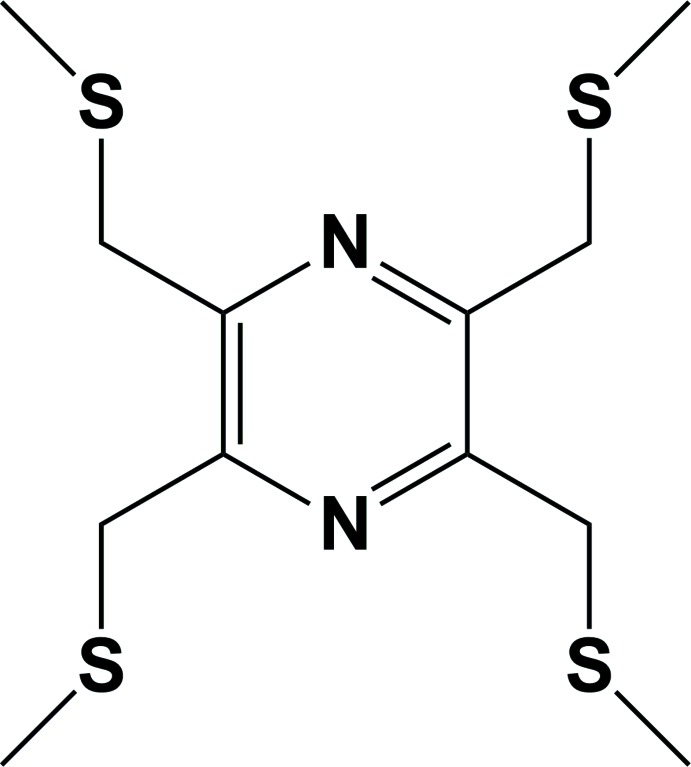


## Experimental   

### Crystal data  

C_12_H_20_N_2_S_4_
*M* *_r_* = 320.54
Triclinic, 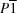

*a* = 6.6773 (6) Å
*b* = 6.9433 (4) Å
*c* = 9.5135 (5) Å
α = 102.635 (6)°
β = 107.539 (5)°
γ = 99.462 (9)°
*V* = 397.61 (5) Å^3^
*Z* = 1
Mo *K*α radiation
μ = 0.58 mm^−1^
*T* = 293 K
0.40 × 0.40 × 0.23 mm

### Data collection  

Stoe AED2 four-circle diffractometer
2960 measured reflections
1478 independent reflections
1283 reflections with *I* > 2σ(*I*)
*R*_int_ = 0.018
3 standard reflections every 60 min intensity decay: 1%

### Refinement  

*R*[*F* ^2^ > 2σ(*F* ^2^)] = 0.032
*wR*(*F* ^2^) = 0.088
*S* = 1.08
1478 reflections
85 parameters
H-atom parameters constrained
Δρ_max_ = 0.24 e Å^−3^
Δρ_min_ = −0.20 e Å^−3^

### 

Data collection: *STADI4* (Stoe & Cie, 1997 ▶); cell refinement: *STADI4*; data reduction: *X-RED* (Stoe & Cie, 1997 ▶); program(s) used to solve structure: *SHELXS97* (Sheldrick, 2008 ▶); program(s) used to refine structure: *SHELXL2013* (Sheldrick, 2008 ▶); molecular graphics: *Mercury* (Macrae *et al.*, 2008 ▶); software used to prepare material for publication: *SHELXL2013*, *PLATON* (Spek, 2009 ▶) and *publCIF* (Westrip, 2010 ▶).

## Supplementary Material

Crystal structure: contains datablock(s) I, Global. DOI: 10.1107/S1600536814011246/hb0006sup1.cif

Structure factors: contains datablock(s) I. DOI: 10.1107/S1600536814011246/hb0006Isup2.hkl

**Fig. 1.**
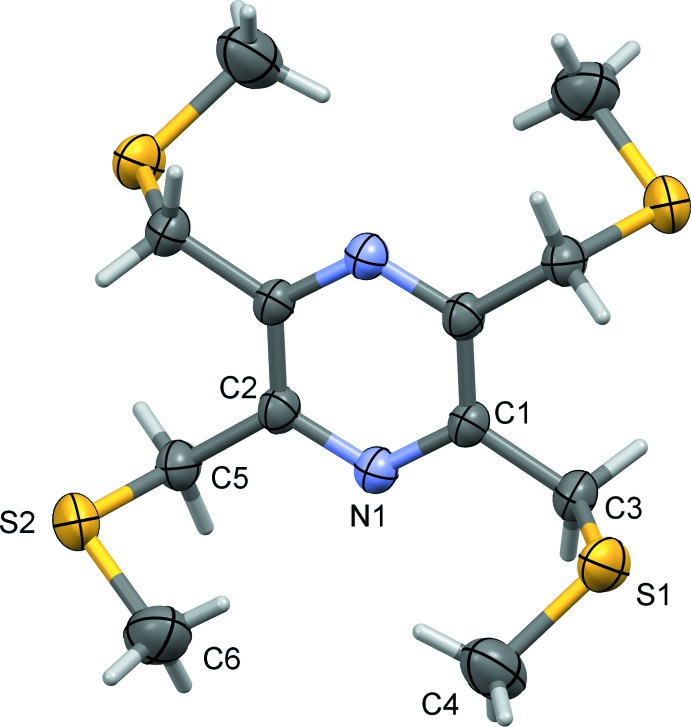
A view of the molecular structure of the title molecule, with atom labelling (unlabelled atoms are generated by inversion symmetry with symmetry code: -x, -y+1, -z + 1). Displacement ellipsoids are drawn at the 50% probability level.

Supporting information file. DOI: 10.1107/S1600536814011246/hb0006Isup3.cml

x y+1 z . DOI: 10.1107/S1600536814011246/hb0006fig1.tif
A view of the mol­ecular structure of the title mol­ecule, with atom labelling (unlabelled atoms are generated by inversion symmetry with symmetry code: −*x*, -*y+1*, −*z* + 1). Displacement ellipsoids are drawn at the 50% probability level.

**Fig. 2.**
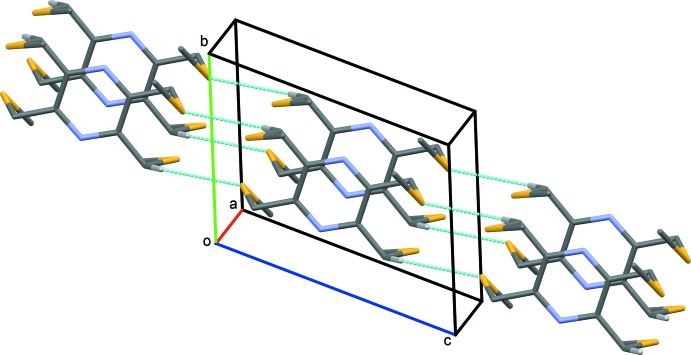
A partial view along the a axis of the crystal packing of the title compound, showing the formation of the C—H···S hydrogen-bonded chains along [001], enclosing R22(12) ring motifs (H atoms not involved in these hydrogen bonds have been omitted for clarity).

Click here for additional data file.a . DOI: 10.1107/S1600536814011246/hb0006fig2.tif
A partial view along the *a* axis of the crystal packing of the title compound, showing the formation of the C—H⋯S hydrogen-bonded chains along [001], enclosing 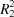
(12) ring motifs (H atoms not involved in these hydrogen bonds have been omitted for clarity).

CCDC reference: 1004261

Additional supporting information:  crystallographic information; 3D view; checkCIF report

## Figures and Tables

**Table 1:** Hydrogen-bond geometry (Å, °)

*D*—H⋯*A*	*D*—H	H⋯*A*	*D*⋯*A*	*D*—H⋯*A*
C3—H3*A*⋯S2^i^	0.97	2.89	3.589 (2)	130
C5—H5*B*⋯S1^ii^	0.97	2.95	3.7395 (19)	139
C5—H5*B*⋯S1^i^	0.97	2.93	3.614 (2)	128

Symmetry codes: (i) 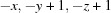; (ii) 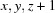.

## supplementary crystallographic information

### S1.1. Synthesis and crystallization

A mixture of sodium methane­thiol­ate (0.94 g, 13 mmol, Fluka 95%) in ethanol (50 ml) was added slowly and drop wise with stirring to a refluxed solution of 2,3,5,6-tetra­kis(bromo­methyl)­pyrazine [Assoumatine & Stoeckli-Evans, 2014], (1.5 g, 3.32 mmol) in ethanol (50 ml). Refluxing and stirring were continued for 4 h. The solvent was removed under reduced pressure and the resultant residue diluted with CH_2_Cl_2_ (200 ml). The organic layer was washed with water (3 × 30 ml) and saturated NaCl (30 ml), then dried over anhydrous MgSO_4_ and evaporated to dryness after filtration. The brown residue obtained was washed with aceto­nitrile till this solvent became colourless, yielding the title compound which was further dried under vacuum [Yield 0.55 g (52%), M.p. 418-419 K]; Rf 0.48 (solvent : CH_2_Cl_2_, eluent : toluene/MeCO_2_Et, 10/1 v/v). Diffusion of an equal volume of ethanol into a concentrated CHCl_3_ (4 ml) solution of the title compound in a 16 mm diameter glass tube yielded suitable yellow plate-like crystals for X-ray diffraction analysis. Spectroscopic data: ^1^H-NMR (CDCl_3_, 400 MHz): δ = 3.97 (s, 8H, Pz—CH_2_—S), 2.13 (s, 12H, S—CH_3_) ppm. ^13^C-NMR (CDCl_3_, 100 MHz): δ = 149.50, 35.89, 15.65 ppm. Anal. for C_12_H_20_N_2_S_4_ (Mr = 320.58 g/mol) Calc. (%): C 44.96; H 6.30; N 8.74; S 40.00. Found (%): C 44.65; H 6.24; N 8.76; S 40.03. MS (EI, 70 eV), m/z (%): 320 ([M^+^], 89.4), 274 (95.2), 257 (50.1), 227 (100), 210 (25.4), 194 (37.9), 181 (54.2), 164 (29.2), 135 (28.1), 97 (23.8). IR (KBr disc, cm^-1^): 2967 w, 2915 w, 1425 ms, 1394 vs, 1311 w, 1248 w, 1218 s, 1120 ms, 988 ms, 903 w, 795 w, 754 w, 720 w, 679 w, 484 w.

### S1.2. Refinement

The H atoms were included in calculated positions and treated as riding atoms: C—H = 0.96 - 0.97 Å with U_iso_(H) = 1.5U_eq_(C-methyl) and = 1.2U_eq_(C) for other H atoms. No absorption correction was applied owing to the irregular shape of the crystal, and as there were no suitable reflections for psi scans.

### Crystal data

**Table:**

C_12_H_20_N_2_S_4_	*Z* = 1
*M**_r_* = 320.54	*F*(000) = 170
Triclinic, *P*1	*D*_x_ = 1.339 Mg m^−^^3^
Hall symbol: -P 1	Mo *K*α radiation, λ = 0.71073 Å
*a* = 6.6773 (6) Å	Cell parameters from 33 reflections
*b* = 6.9433 (4) Å	θ = 14.2–18.8°
*c* = 9.5135 (5) Å	µ = 0.58 mm^−^^1^
α = 102.635 (6)°	*T* = 293 K
β = 107.539 (5)°	Plate, yellow
γ = 99.462 (9)°	0.40 × 0.40 × 0.23 mm
*V* = 397.61 (5) Å^3^	

### Data collection

**Table:**

Stoe AED2 four-circle diffractometer	*R*_int_ = 0.018
Radiation source: fine-focus sealed tube	θ_max_ = 25.5°, θ_min_ = 2.3°
Graphite monochromator	*h* = −8→7
2θ/ω scans	*k* = −8→8
2960 measured reflections	*l* = 0→11
1478 independent reflections	3 standard reflections every 60 min
1283 reflections with *I* > 2σ(*I*)	intensity decay: 1%

### Refinement

**Table:**

Refinement on *F*^2^	Secondary atom site location: difference Fourier map
Least-squares matrix: full	Hydrogen site location: inferred from neighbouring sites
*R*[*F*^2^ > 2σ(*F*^2^)] = 0.032	H-atom parameters constrained
*wR*(*F*^2^) = 0.088	*w* = 1/[σ^2^(*F*_o_^2^) + (0.0451*P*)^2^ + 0.1143*P*] where *P* = (*F*_o_^2^ + 2*F*_c_^2^)/3
*S* = 1.08	(Δ/σ)_max_ < 0.001
1478 reflections	Δρ_max_ = 0.24 e Å^−^^3^
85 parameters	Δρ_min_ = −0.20 e Å^−^^3^
0 restraints	Extinction correction: *SHELXL2013* (Sheldrick, 2008), Fc^*^=kFc[1+0.001xFc^2^λ^3^/sin(2θ)]^-1/4^
Primary atom site location: structure-invariant direct methods	Extinction coefficient: 0.064 (11)

### Special details

**Table:**

Geometry. Bond distances, angles *etc*. have been calculated using the rounded fractional coordinates. All su's are estimated from the variances of the (full) variance-covariance matrix. The cell e.s.d.'s are taken into account in the estimation of distances, angles and torsion angles

### Fractional atomic coordinates and isotropic or equivalent isotropic displacement parameters (Å^2^)

**Table:**

	*x*	*y*	*z*	*U*_iso_*/*U*_eq_	
S1	0.10172 (10)	0.33963 (9)	0.10899 (6)	0.0509 (2)	
S2	0.37011 (9)	0.28482 (9)	0.80525 (6)	0.0515 (2)	
N1	−0.0169 (2)	0.2930 (2)	0.44718 (16)	0.0326 (4)	
C1	−0.0580 (3)	0.4056 (3)	0.34870 (19)	0.0311 (5)	
C2	0.0402 (3)	0.3854 (3)	0.59786 (19)	0.0313 (5)	
C3	−0.1194 (3)	0.2955 (3)	0.1806 (2)	0.0390 (6)	
C4	0.2772 (5)	0.2016 (4)	0.2027 (3)	0.0684 (10)	
C5	0.0843 (3)	0.2519 (3)	0.7029 (2)	0.0376 (6)	
C6	0.4534 (4)	0.1829 (4)	0.6484 (3)	0.0665 (9)	
H3A	−0.23980	0.33950	0.12120	0.0470*	
H3B	−0.16800	0.15040	0.16540	0.0470*	
H4A	0.33380	0.26610	0.31160	0.1030*	
H4B	0.39480	0.20040	0.16450	0.1030*	
H4C	0.19730	0.06420	0.18230	0.1030*	
H5A	0.02160	0.11060	0.64270	0.0450*	
H5B	0.01290	0.28160	0.77720	0.0450*	
H6A	0.36700	0.04660	0.59450	0.1000*	
H6B	0.60330	0.18130	0.68720	0.1000*	
H6C	0.43450	0.26620	0.57940	0.1000*	

### Atomic displacement parameters (Å^2^)

**Table:**

	*U*^11^	*U*^22^	*U*^33^	*U*^12^	*U*^13^	*U*^23^
S1	0.0731 (4)	0.0608 (4)	0.0394 (3)	0.0318 (3)	0.0335 (3)	0.0242 (3)
S2	0.0568 (4)	0.0584 (4)	0.0352 (3)	0.0256 (3)	0.0050 (2)	0.0128 (2)
N1	0.0386 (8)	0.0332 (8)	0.0285 (7)	0.0114 (6)	0.0133 (6)	0.0097 (6)
C1	0.0355 (9)	0.0350 (9)	0.0252 (8)	0.0112 (7)	0.0123 (7)	0.0092 (7)
C2	0.0340 (9)	0.0371 (9)	0.0272 (8)	0.0112 (7)	0.0134 (7)	0.0123 (7)
C3	0.0492 (11)	0.0408 (10)	0.0265 (9)	0.0141 (8)	0.0124 (8)	0.0076 (8)
C4	0.0750 (17)	0.0855 (19)	0.0741 (17)	0.0467 (15)	0.0399 (14)	0.0415 (15)
C5	0.0493 (11)	0.0384 (10)	0.0307 (9)	0.0154 (8)	0.0164 (8)	0.0145 (8)
C6	0.0570 (15)	0.0784 (18)	0.0633 (16)	0.0307 (13)	0.0207 (12)	0.0090 (13)

### Geometric parameters (Å, º)

**Table:**

S1—C3	1.813 (2)	C3—H3B	0.9700
S1—C4	1.789 (3)	C4—H4A	0.9600
S2—C5	1.812 (2)	C4—H4B	0.9600
S2—C6	1.790 (3)	C4—H4C	0.9600
N1—C1	1.342 (2)	C5—H5A	0.9700
N1—C2	1.342 (2)	C5—H5B	0.9700
C1—C3	1.509 (2)	C6—H6A	0.9600
C1—C2^i^	1.401 (3)	C6—H6B	0.9600
C2—C5	1.504 (3)	C6—H6C	0.9600
C3—H3A	0.9700		
			
C3—S1—C4	101.45 (13)	S1—C4—H4B	109.00
C5—S2—C6	100.09 (11)	S1—C4—H4C	109.00
C1—N1—C2	118.31 (16)	H4A—C4—H4B	109.00
N1—C1—C3	116.40 (17)	H4A—C4—H4C	109.00
N1—C1—C2^i^	120.78 (15)	H4B—C4—H4C	109.00
C2^i^—C1—C3	122.82 (17)	S2—C5—H5A	109.00
N1—C2—C5	116.03 (17)	S2—C5—H5B	109.00
N1—C2—C1^i^	120.91 (17)	C2—C5—H5A	109.00
C1^i^—C2—C5	123.05 (15)	C2—C5—H5B	109.00
S1—C3—C1	113.18 (14)	H5A—C5—H5B	108.00
S2—C5—C2	113.56 (15)	S2—C6—H6A	109.00
S1—C3—H3A	109.00	S2—C6—H6B	109.00
S1—C3—H3B	109.00	S2—C6—H6C	109.00
C1—C3—H3A	109.00	H6A—C6—H6B	110.00
C1—C3—H3B	109.00	H6A—C6—H6C	109.00
H3A—C3—H3B	108.00	H6B—C6—H6C	109.00
S1—C4—H4A	109.00		
			
C4—S1—C3—C1	70.47 (18)	C2^i^—C1—C3—S1	77.1 (2)
C6—S2—C5—C2	−67.65 (17)	N1—C1—C2^i^—N1^i^	−0.5 (3)
C2—N1—C1—C3	179.41 (18)	N1—C1—C2^i^—C5^i^	−180.00 (19)
C2—N1—C1—C2^i^	0.4 (3)	C3—C1—C2^i^—N1^i^	−179.35 (18)
C1—N1—C2—C5	179.98 (18)	C3—C1—C2^i^—C5^i^	1.1 (3)
C1—N1—C2—C1^i^	−0.4 (3)	N1—C2—C5—S2	103.98 (18)
N1—C1—C3—S1	−101.84 (19)	C1^i^—C2—C5—S2	−75.6 (2)

Symmetry code: (i) −*x*, −*y*+1, −*z*+1.

### Hydrogen-bond geometry (Å, º)

**Table:**

*D*—H···*A*	*D*—H	H···*A*	*D*···*A*	*D*—H···*A*
C3—H3*A*···S2^i^	0.97	2.89	3.589 (2)	130
C5—H5*B*···S1^ii^	0.97	2.95	3.7395 (19)	139
C5—H5*B*···S1^i^	0.97	2.93	3.614 (2)	128

Symmetry codes: (i) −*x*, −*y*+1, −*z*+1; (ii) *x*, *y*, *z*+1.
