# Supplementary figures and images for: Crystal structure of 2,3,5,6-tetra­kis[(methyl­sulfan­yl)meth­yl]pyrazine
Source: Acta Crystallogr Sect E Struct Rep Online. 2014 Aug 1;70(Pt 9):o887–8. doi: 10.1107/S1600536814011246 (PMC4186201; doi:10.1107/S1600536814011246)

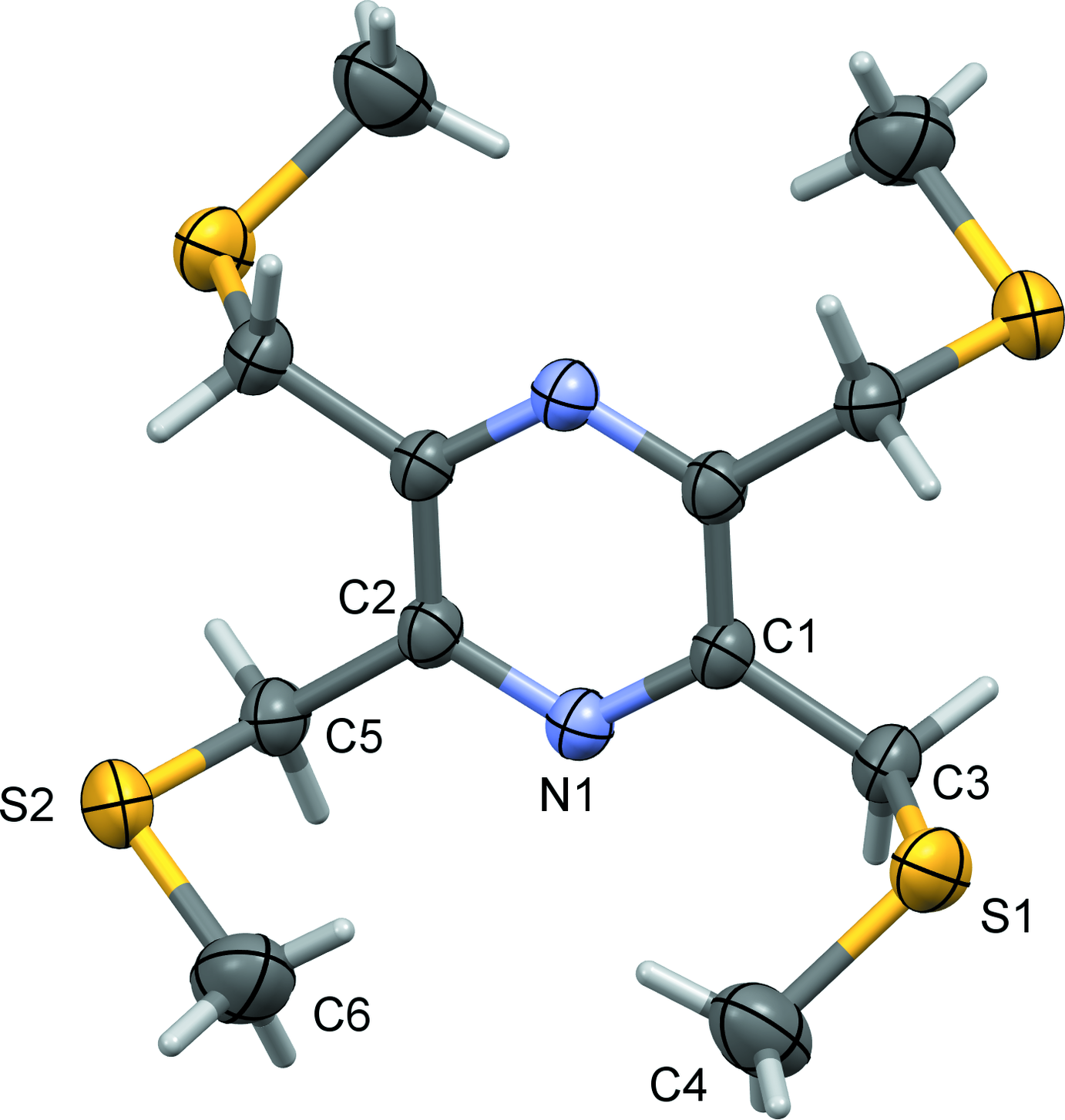

Supplement: Supplementary file 4 [file e-70-0o887-fig1.tif]

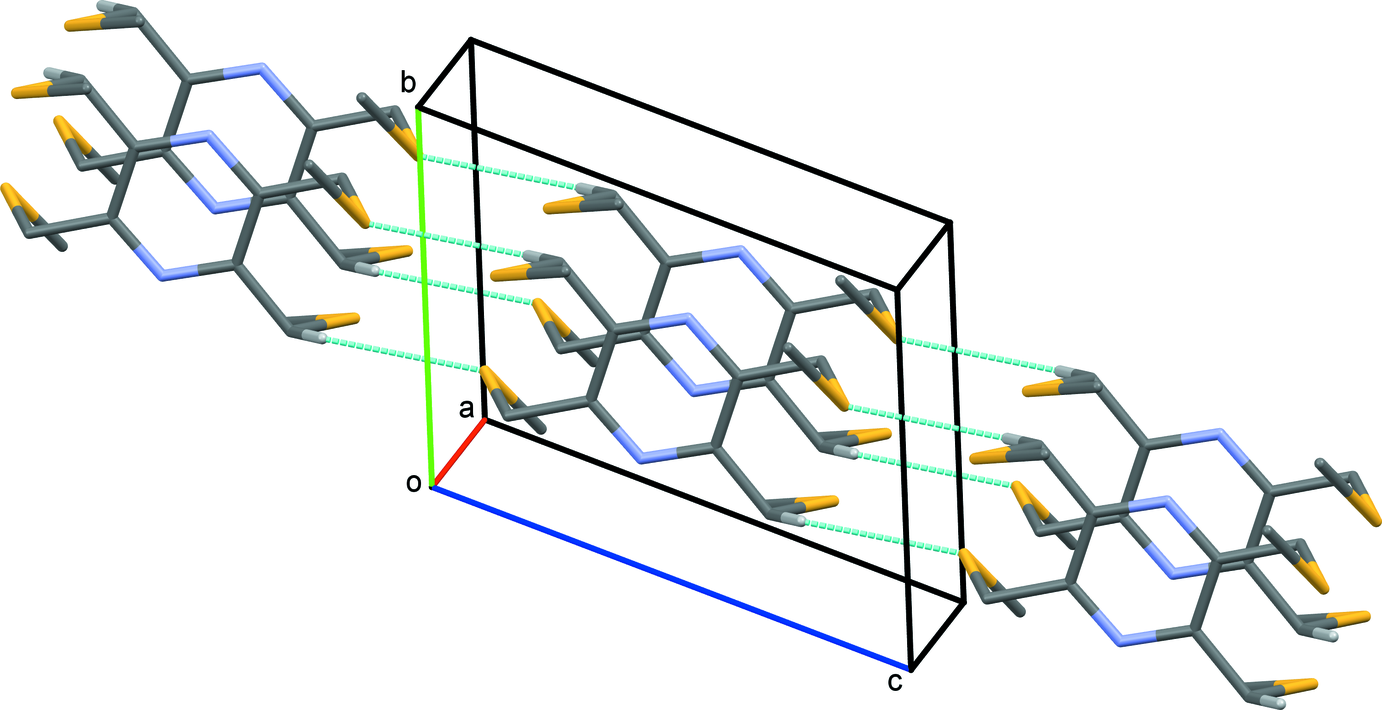

Supplement: Supplementary file 5 [file e-70-0o887-fig2.tif]
